# Optical Clearing in Dense Connective Tissues to Visualize Cellular Connectivity *In Situ*

**DOI:** 10.1371/journal.pone.0116662

**Published:** 2015-01-12

**Authors:** Sarah Calve, Andrew Ready, Christopher Huppenbauer, Russell Main, Corey P. Neu

**Affiliations:** 1 Weldon School of Biomedical Engineering, Purdue University, West Lafayette, Indiana, United States of America; 2 W. Nuhsbaum, Inc., McHenry, Illinois, United States of America; 3 Department of Basic Medical Sciences, Purdue University, West Lafayette, Indiana, United States of America; Medical College of Georgia, UNITED STATES

## Abstract

Visualizing the three-dimensional morphology and spatial patterning of cells embedded deep within dense connective tissues of the musculoskeletal system has been possible only by utilizing destructive techniques. Here we utilize fructose-based clearing solutions to image cell connectivity and deep tissue-scale patterning *in situ* by standard confocal microscopy. Optical clearing takes advantage of refractive index matching of tissue and the embedding medium to visualize light transmission through a broad range of bovine and whole mount murine tissues, including cartilage, bone, and ligament, of the head and hindlimb. Using non-destructive methods, we show for the first time intercellular chondrocyte connections throughout the bulk of cartilage, and we reveal *in situ* patterns of osteocyte processes and the lacunar-canalicular system deep within mineralized cortical bone. Optical clearing of connective tissues is expected to find broad application for the study of cell responses in normal physiology and disease pathology.

## Introduction

With the advent of confocal and multi-photon microscopy, the ability to characterize cellular morphology deep within tissues has prompted the further refinement of clearing methodologies that aim to better match the index of refraction of tissues and the fluid in which they are immersed. This is typically accomplished by equilibrating specimens in aqueous or organic reagents that have indexes of refraction close to that of biological tissue [1, 2] or through the removal of light-scattering lipids [3, 4]. Advances in optical clearing have predominantly centered around the fields of embryology and neurology [1–6]. Recently these techniques have been adapted for studies investigating adult tissues beyond the brain, including large intestine [7], lung [8], heart [9], skin [10], spinal cord and peripheral nerve [4, 11], as well as tissue engineered constructs [12]. However, the use of optical clearing for the visualization of cellular morphology, spatial patterning, or connectivity within the musculoskeletal system has not been reported.

The tissues of the musculoskeletal system are predominantly composed of dense extracellular matrix (ECM) networks and mineral (in the case of bone), which limit penetration of light and increase scattering in microscopy studies. Consequently, non-destructive investigations into cell-cell and cell-ECM interactions have been limited to the surface of these tissues, and yet recent studies suggest that cells deeply embedded within musculoskeletal tissues profoundly influence normal physiology and the pathogenesis of numerous diseases, for example osteoarthritis [13] and osteoporosis [14]. Visualization and study of sub-surface or deep cells that have different phenotypes and morphology than those near the surface has historically required mechanical sectioning, which can introduce artifacts and is destructive to the native tissue. Without the ability to non-destructively probe and image the 3D morphology of musculoskeletal cells *in situ*, we are not yet able to address many elementary unresolved issues, including a) the extent that chondrocytes communicate with adjacent cells, b) patterns of cellular connections across tissue interfaces (e.g. entheses and myotendinous junctions) that may facilitate mechanical/chemical crosstalk, c) how baseline cell distributions within tissues influence normal physiology, and d) how deviations from the norm define specific disease states and the progression toward regeneration and repair.

We explored the possibility that optical clearing of dense connective tissues could reveal unique microstructural and cellular characteristics that are not easily observed or quantified using standard microscopy or histomorphometry techniques. The main objective of this study was to determine the effect of optical clearing on cellular morphology within bovine connective tissues, including cartilage, bone, meniscus, and ligament, and whole mount murine tissues of the head and hindlimb. We reveal the utility of SeeDB [1], a fructose-based clearing agent that requires minimal processing during both tissue harvest and image analysis, for characterizing the 3D distribution of cells in connective tissue rich specimens, a representative biological example that highlights our ability visualize cellular structures within tissues *in situ*. SeeDB is a simple water-based optical clearing agent that takes advantage of saturated fructose solutions having a refractive index close to that of fixed tissue (*n* = 1.49) and permits imaging to depths greater than 2,000 μm using conventional confocal microscopy in brain tissue [1]. The combination of optical clearing and membrane-localized fluorophores enabled us to probe deep within musculoskeletal tissues *in situ* to confirm the presence of intercellular connections between adjacent chondrocytes and resolve the intricate dendritic processes that osteocytes use for intercellular communication while embedded within mineralized cortical bone.

## Materials and Methods

### Animal care and tissue harvest

Multiple tissues from bovine and murine sources were used in optical clearing experiments. Musculoskeletal tissues were harvested from juvenile bovine knee (stifle) joints obtained from a local United States Department of Agriculture (USDA) regulated abattoir (Dutch Valley Foods, Holland, IL) within 24 hours of slaughter. As these joints were classified as non-edible food items, no additional regulatory compliance was necessary. Joints (n ≥ 3) were opened under aseptic conditions to expose the tibial and femoral condyles and the trochlear groove. Osteochondral (cartilage-bone) plugs were removed from the load-bearing region of the medial condyle using a 5 mm diameter coring reamer [15]. Joint tissue sections were likewise harvested from the medial meniscus, synovium, and anterior cruciate ligament, and separately from the quadriceps muscle and tendon. Tissues were immediately fixed in 4% paraformaldehyde (PFA) diluted in phosphate buffered saline (PBS) for 1–2 days at 4**°**C with gentle rocking then rinsed at least 2× 1 hour with PBS at room temperature with gentle rocking. Tissues were trimmed to specimens 2.5–3 mm thick before beginning the clearing process (see below).

Young adult mice heterozygous for either Pax3-Cre:R26ZsGreen [16, 17] or mT/mG [18] were utilized for bulk tissue optical clearing (derived from animals obtained from The Jackson Laboratory). All murine experiments were approved by the Purdue Animal Care and Use Committee (PACUC; protocols 1305000861 and 1209000723). The PACUC ensures that all animal programs, procedures, and facilities at Purdue University adhere to the policies, recommendations, guidelines, and regulations of the USDA and the United States Public Health Service (USPHS) in accordance with the Animal Welfare Act and Purdue’s Animal Welfare Assurance. Mice were euthanized via CO_2_ inhalation, which was confirmed by cervical dislocation. Like bovine tissues, murine organ and multi-tissue systems were immediately harvested and fixed in 4% PFA for 1–2 days at 4°C, and then rinsed with PBS. Subsequent fluorescent labeling and clearing of fixed samples were carried out immediately or after storage at 4**°**C for no more than 7 days.

### Fluorescent labeling of fixed tissues

Fixed bovine tissues were stained with AlexaFluor 488 conjugated phalloidin (1:100), SP-DiIC_18_(3) (1:250; a DiI analogue that has enhanced solubility in aqueous solutions due to sulfonate groups) or AlexaFluor 488 conjugated wheat germ agglutinin (WGA; 1:200) diluted in PBS, and counterstained with Hoescht 34580 (1:500) for 24–48 hours at 4**°**C with gentle rocking. All staining reagents were obtained from Life Technologies. Tissues were rinsed 3× 1 hour with PBS at room temperature and imaged using confocal microscopy before or after clearing.

### Optical Clearing

Tissues were cleared following Ke et al., 2013 [1]. Fructose solutions of varying concentrations (20%, 40%, 60%, 80%, 100% and 115% wt/vol) were generated by dissolving D-(-)-fructose (JT Baker, Center Valley, PA) in milliQ water with 0.5% α-thioglycerol (Sigma-Aldrich St. Louis, MO) to prevent browning. Tissues were equilibrated to increasing concentrations of fructose by incubating in each formulation for at least 24 hours under gentle rocking at room temperature. Unclearing, or reversal to PBS, was performed by equilibrating samples in fructose solutions of decreasing concentrations under the same conditions for clearing.

### Imaging

Using a stereo dissecting microscope (Leica M80, Wetzlar Germany), light transmission was visualized through the bulk bovine and murine tissues. Images were acquired with front and back lighting to visualize bovine tissues placed on a printed grid pattern (spacing = 2.1 mm).

The comparison of signal penetration was carried out on a Nikon A1Rsi inverted confocal microscope using a 20× multi-immersion objective (Plan Fluor, NA = 0.75, working distance ≈ 300 µm) with four available excitation laser wavelengths (405, 488, 555, 633 nm). Specimens were imaged with a 3× optical zoom and 2× frame average. For control samples, water was the immersion medium and cleared samples utilized oil with a refractive index of 1.515. Imaging parameters: field of view = 211.9 × 211.9 µm^2^; matrix = 512 × 512 pixels^2^, Δ*z* = 1.0 μm.

A Leica TCS LSI-III Confocal was used to image the cleared head of an mT/mG mouse in which all tissues remained intact except for the overlying skin. The sample was placed in a standard tissue culture dish with minimal amount of clearing solution. The area to be imaged was faced upwards toward the objective lens and no further dissection was required as the samples were non-invasively imaged. An initial lambda scan was performed to determine the optimal emission spectra to set the spectral prism. Tissues were excited with 561 nm laser set at 100% on AOFT for maximum penetration and scanned with a frame average of 3 or 4×. Airy unit sizes were varied to achieve the best image quality and deeper penetration into sample required a larger confocal pinhole size.

Osteocyte processes within the distal femur from an mT/mG mouse were imaged using a Zeiss LSM 710 equipped with a 25× LD LCI Plan-Apochromat multi-immersion lens (NA = 0.8, immersion oil *n* = 1.518). Imaging parameters: excitation wavelength = 561 nm (tdTomato); field of view = 113.4 × 113.4 µm^2^; matrix = 1024 × 1024 pixels^2^; Δ*z* = 1.0 µm.

### Image Processing

Confocal stacks were rendered in 3D using FIJI (NIH). Figures were assembled by taking 2D slices from stacks or snapshots of 3D rendered image volumes and arranged using Adobe Photoshop and Illustrator.

## Results

**Figure 1:**
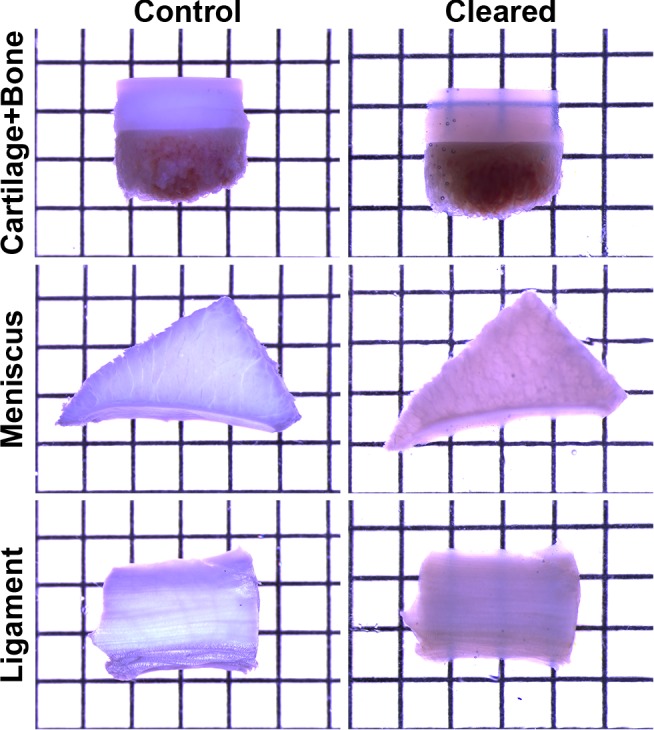
Optical clearing of ECM-rich bovine musculoskeletal tissues. Equilibration to concentrated fructose solutions that closely match the refractive index of biological tissue substantially enhances the macroscopic transmission of light though 2.5 mm thick samples of cartilage (top row) and ligament (bottom row). While the transparency of light was greater in cleared meniscus (middle row, see also Fig. 2), the background grid pattern was not easily visualized. Control and cleared samples were imaged using the same acquisition parameters on a Leica MZ80 stereomicroscope. Grid spacing = 2.1 mm.

To determine the utility of optical clearing for musculoskeletal tissues, we first tested the extent that clearing enhanced light transmission at the macroscopic level. When 2.5 mm-thick slices of cartilage (top row) and ligament (bottom row) were equilibrated in concentrated fructose solutions, the specimens became noticeably more transparent as evidenced by the ability to resolve grid patterns placed behind the specimen (Fig. 1). While the transmission of light was greater in the meniscus after clearing, the grid could not be visualized (middle row, Fig. 1). Transmission of light through synovium and tendon, and to a lesser extent muscle, was also observed (S1 Fig.).

**Figure 2:**
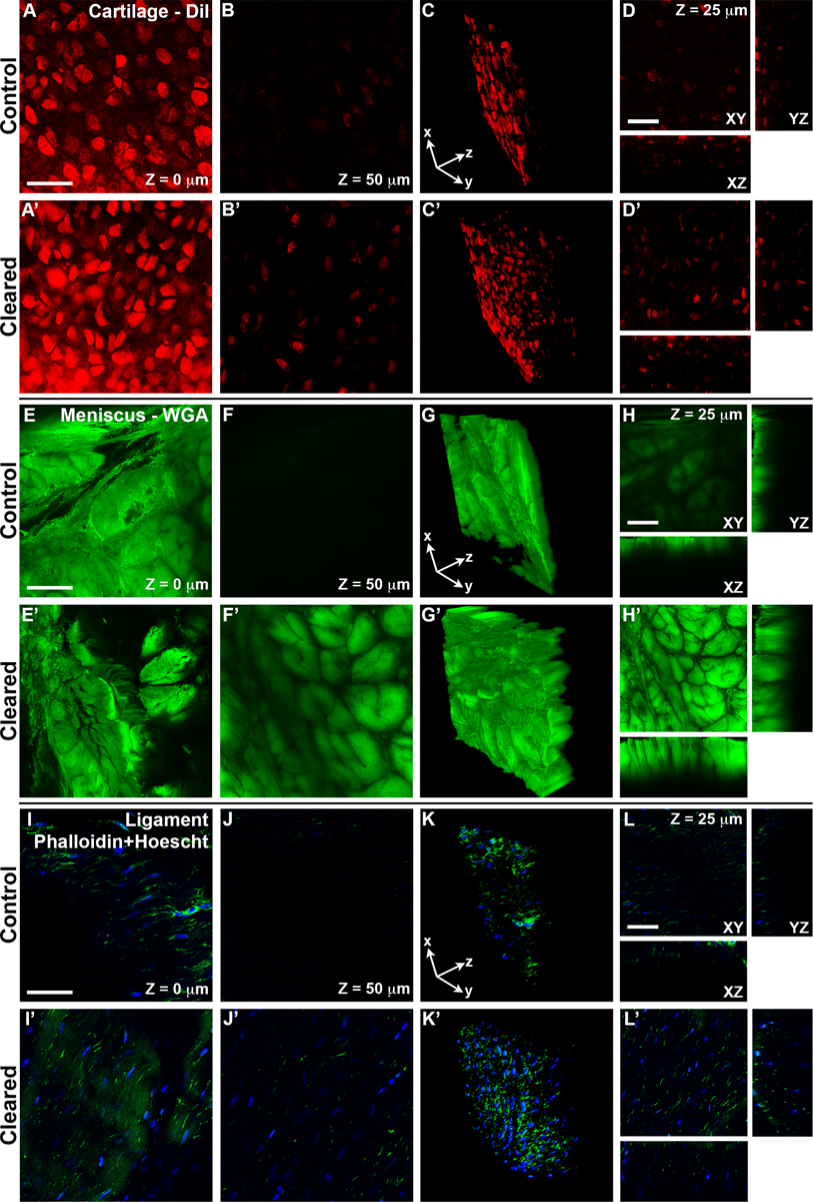
Confocal visualization of cellular and tissue architecture is enhanced at greater depths after optical clearing. **A—D’**: Bovine articular cartilage was stained with DiI (to label the plasma membrane). In control specimens (A—D), fluorescence intensity greatly diminished 50 μm into the sample, highlighted by 3D rendering (C) and orthogonal sections (D) of a 212 μm × 212 μm × 100 μm (*x* × *y* × *z*) *z*-stack. After clearing (A’—D’), chondrocytes defined by DiI staining could be imaged through the entire representative volume element (see Fig. 3 for entire volume). **E—H’**: Meniscus was stained with WGA to label glycoconjugates, revealing the architecture of the dense ECM at depths greater than 50 μm after clearing (F’). **I—L’**: Ligament was stained with phalloidin (green) and Hoescht 34580 (blue) to label F-actin and cell nuclei respectively. As with cartilage and meniscus, substantial increases in total depth imaged was observed after clearing (I’—L’), particularly evident in the 3D rendering (K’). Specimens were imaged using a Nikon A1R microscope, with a PlanFluor 20× multi-immersion objective, NA = 0.75, 3× optical zoom and 2× frame average. For control samples, water was the immersion medium and cleared samples utilized oil with a refractive index of 1.515. Image dimensions: 512 × 512 pixels^2^, Δ*z* = 1.0 μm. Stacks were rendered in 3D using FIJI. Bars in A, D, E, H, I and L = 50 μm.

**Figure 3:**
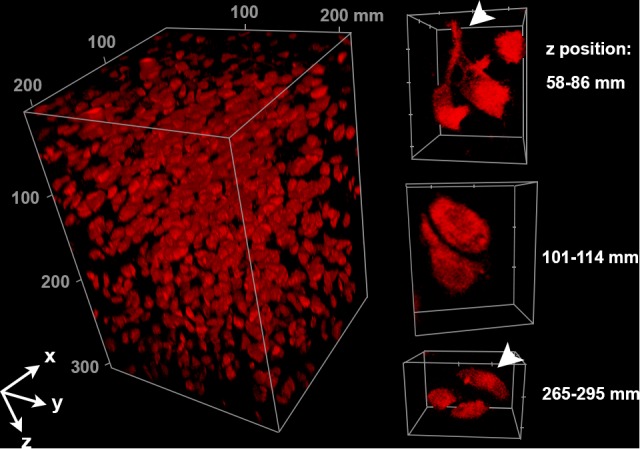
High resolution imaging of cartilage reveals evidence of connectivity between individual chondrocytes. Interactions between individual chondrocytes within bovine articular cartilage can be resolved throughout the entire 307 μm–thick *z*-stack (arrowheads). The same *z*-stack shown in Fig. 2C’was bleached corrected and rendered in 3D using FIJI. The top of the volume was imaged just below the articular surface and goes deeper into the cartilage as *z* increases. Cartilage was stained with DiI, cleared with SeeDB and imaged using a Nikon A1R microscope, with a PlanFluor 20× multi-immersion objective, NA = 0.75, 3× confocal zoom. The depth of the image stack was limited by the working distance of the objective. Tick marks on isolated cell volumes = 10 μm. See also S1–S4Movies.

We next utilized confocal microscopy to determine how SeeDB-based clearing affected specimen visualization at the cellular level. A current bottleneck in the adaptation of clearing techniques for whole-mount labeling of specific tissue components is the slow diffusion of high molecular weight primary and secondary antibodies (MW ≈ 150 kDa for unconjugated IgG). Therefore, to maximize signal penetration, we specifically chose fluorescently-labeled probes of low MW. Starting at the specimen surface, representative volume elements 100 μm thick were acquired at 20× using standard confocal microscopy before and after clearing. Control specimens were placed in PBS and imaged using water immersion (*n* = 1.33). After clearing, specimens were suspended in the 115% fructose solution (*n* = 1.49) and imaged using oil immersion (*n* = 1.518). Due to the inherent differences in imaging with water versus oil immersion, it was not feasible to keep all imaging parameters consistent. Nevertheless, the same laser power, pixel dwell time, and *x*, *y* and *z*-dimensions were used. To emphasize the enhancement of signal intensity due to optical clearing, images were left unprocessed (i.e. no bleach correction or image filtering) except for using FIJI to equilibrate intensity histograms of the stacks taken before and after clearing, based on the fifth *z*-slice from the tissue surface.

Chondrocytes within articular cartilage were labeled using DiI-SP (MW = 1.1 kDa), a lipophilic membrane probe. In control specimens, signal intensity was greatly diminished 50 μm into the sample (Fig. 2A—D), whereas after clearing, chondrocytes could be visualized through the entire 100 μm representative volume element (Fig. 2A’—D’). Meniscus, ligament and cartilage were stained with AlexaFluor 488-conjugated wheat germ agglutinin (WGA; MW = 38 kDa), a lectin that binds to glycoproteins, to visualize ECM organization [19] (Figs. 2E—H’; S2A—H’). Control WGA-stained tissues showed reduced light penetration at 25 μm and little to no signal at 50 μm (Fig. 2F, H; S2B, D, F, H Fig.). After clearing with SeeDB, the architecture of the dense ECM in meniscus and ligament could be resolved at depths greater than 50 μm (Fig. 2E—H’; S2A—D’ Fig.). Fibroblasts within ligament and cartilage were stained with AlexaFluor 488-conjugated phalloidin (green; MW = 1.3 kDa) and Hoescht 34580 (blue; MW = 0.6 kDa) to label F-actin and nuclei respectively (Fig. 2 I—L’; S2I—L’ Fig.). In control ligament and cartilage, cells could not be discerned at a depth of 50 μm (Fig. 2J, S2J Fig.) As with DiI-stained cartilage, phalloidin-labeled cells in cleared ligament and cartilage were visible through the 100 μm-thick representative volume element (Fig. 2K—L’; S2K—L’ Fig.). Autofluorescence in the 488 channel became more prominent after clearing (Fig. 2I’); however, it was mainly restricted to the surface. These data indicate that the imaging of cells and ECM within musculoskeletal tissues can be enhanced at least two-fold by simple equilibration in fructose solutions without any additional acquisition or post-processing enhancements.

The representative volume element containing DiI-SP-stained chondrocytes in 2C’ was taken from a larger 307 μm-thick *z*-stack. When bleach corrected, interactions between DiI-SP labeled chondrocytes could be clearly resolved through the full depth, as we were limited by the working distance of our objectives. At 60 μm and 270 μm away from the articular surface, neighboring chondrocytes could be seen making putative connections (Fig. 3 and S1–S4 Movies).

**Figure 4:**
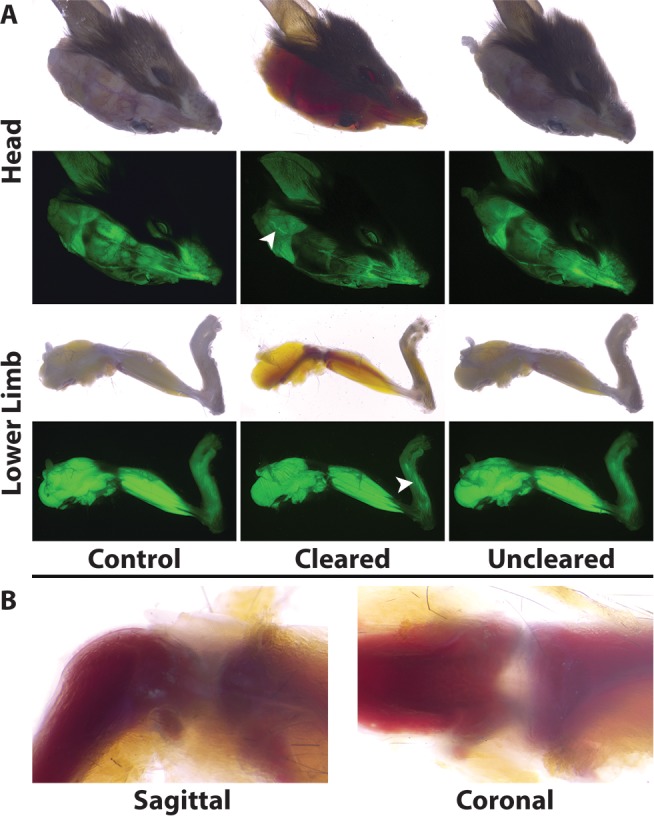
Fructose-based clearing enables the delineation of tissue interactions within the murine head and limb. **A:** Reversible clearing maintained tissue and fluorescence integrity in Pax3-Cre:R26ZsGreen heterozygotes. The calvaria became transparent revealing the distinct architecture of the nasal cavity along with the caudal vasculature (top row, arrowhead). Clearing of skin covering the distal portion of the lower limb allows for the greater resolution of the foot musculature (bottom row, arrowhead). **B:** In addition, tissue interactions of the knee joint, including contact of the femur and tibia, become evident once the ligaments and fascia are rendered transparent.

When intact murine heads and hindlimbs were cleared, structures normally hidden by dense connective tissues (i.e. calvaria and ligaments/fascia) could be visualized at the macroscopic scale (Fig. 4A). To also assess if the resolution of fluorescent structures was enhanced, tissues from Pax3-Cre:R26ZsGreen mice were used. In this mouse line, all descendants of Pax3-expressing cells are GFP+ including skeletal muscle, neural crest and a small subset of endothelial cells [20]. While some structures became clearer in the concentrated fructose solution, for example large nerves in the head (arrowhead, top row, Fig. 4A) and musculature as seen through the skin of the foot (arrowhead, bottom row, Fig. 4A), the strong fluorescent signal, combined with widefield microscopy, interfered with visualization of fine structures in these bulk tissues.

**Figure 5:**
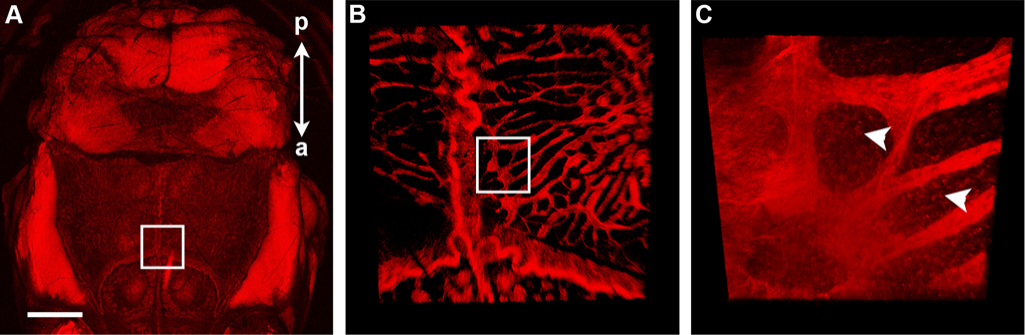
Multiscale visualization of cleared tissues in the murine head. Clearing of the calvaria in an mT/mG mouse revealed the vasculature within the skull and the underlying meninges. Individual osteocytes delineated by membrane-localized tdTomato, could be identified (arrowheads, C). All images taken at a total optical magnification of 6.2× and 512 × 512 pixel^2^ with a Leica LSI confocal microscope. A: Maximum intensity projection of 100 slices taken at 1× confocal zoom, field of view = 31.2 × 31.2 mm^2^, Δz = 123.8 μm, 4× frame average. Bar = 5 mm. B: Confocal zoom of box in (A). 3D rendering of maximum intensity projection of 12 slices, field of view = 11.8 × 11.8 mm^2^, Δ*z* = 45.8 μm, 3× frame average. C: Zoom of box in (B). 3D rendering of maximum intensity projection of 12 slices, field of view = 582 × 582 μm^2^, Δ*z* = 22.9 μm, 3× frame average. The skin was removed from the head prior to clearing and imaging. The anterior—posterior axis is indicated in (A).

In contrast, we were able to visualize the morphology of cleared bone at multiple scales utilizing tissues from the mT/mG mouse line, in which all cells express membrane-localized tdTomato [18]. Using a Leica LSI system, a large-scale true point scanning spectral confocal, we were able to scan the overview of a cleared mouse head and in to the finer details without changing objectives zoom. The skin was removed from the intact head and then scanned with out any further dissection. Clearing with SeeDB, in combination with the unique design of the microscope, allowed visualization of the vasculature within the skull and the interface of the skull and meninges (Fig. 5A). To image deeper with greater confocal zoom the pinhole was opened wider to three airy units, enabling the higher resolution of structure in this undissected mouse head, highlighting the vasculature (Fig. 5B) and osteocyte distribution (arrowheads, Fig. 5C).

**Figure 6:**
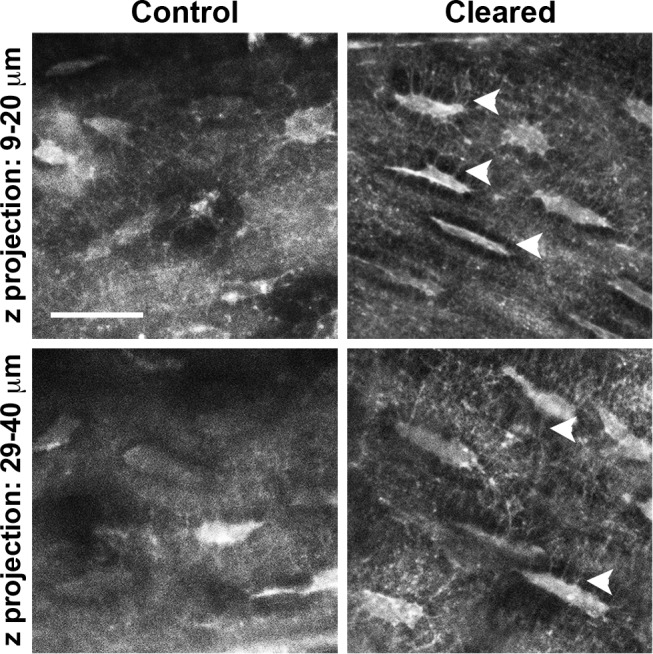
Visualization of osteocyte processes within intact bone. The distal epiphysis of a femur from an mTmG mouse was imaged after equilibration in either PBS or SeeDB. Z-projections of the cleared bone, encompassing 10 μm, revealed distinct cytoplasmic extensions at both 9 and 29 μm below the surface of the bone (arrowheads) whereas only faint outlines could be discerned in the control after equivalent image processing. The femur was kept intact (i.e. without sectioning or fracturing) and imaged through the periosteum using a Zeiss 710 confocal microscope with a 25× multi-immersion lens, NA = 0.8, 3× confocal zoom. Images were bleach corrected using FIJI, and then *z*-projections were made without any additional post-processing. Bar = 20 μm.

One of the limitations of the Leica LSI is that the upright objective utilizes air immersion, preventing the use of higher power objectives. Therefore, to better visualize osteocytes *in situ*, we isolated an intact femur from the mT/mG mouse line and compared cell morphology in uncleared and cleared specimens. After clearing, the fine cytoplasmic processes of osteocytes in the distal epiphysis of the femur could be imaged to 40 μm below the bone surface. In cleared samples, osteocyte dendritic processes were plainly visible extending from the osteocyte cell body, through the pericellular space to the lacunar wall, and continuing through the fine intercellular canaliculi coursing through the mineralized bone tissue. The morphologic details of osteocytes in uncleared bone were difficult to discern (Fig. 6). Apart from bleach correction and brightness/contrast adjustment, these images were not subject to any additional post-processing.

## Discussion

A number of optical clearing methods that facilitate the imaging of cellular morphology deep within tissues have been developed; however the majority are not without limitations: CLARITY requires specialized equipment [3]; ClearT is based on formamide, a highly corrosive teratogen [2]; 3DISCO is not compatible with lipid based dyes or investigations of membrane-bound molecules of interest [4]; Sca*l*e and BABB induce significant changes in specimen volume [5, 6]; and no clearing agents yet permit deep imaging of live, viable cells in native tissue. SeeDB relies upon basic reagents, does not influence specimen size or integrity and clearing of tissues takes less than a week, whereas other methods such as CLARITY and Sca*l*e can require a minimum of 2 weeks. The initial study by Ke et al. described how SeeDB enhances light transmission in embryonic tissue and adult brain, quantifying the advantage of SeeDB over Sca*l*e and BABB in maintaining specimen fluorescence and geometry [1]. Herein, we show the expanded utility of this simple agent by demonstrating that tissues of the musculoskeletal system can be cleared using SeeDB to reveal interactions between cells embedded within a dense, and even mineralized, ECM.

Certain types of optical clearing methodologies have been previously reported for tendon [21], bone [22] and skin [23]; however, the aim of these studies was to enhance in vivo imaging and phototherapy through these cleared tissues rather than characterize the cells or ECM within them [24]. The agents utilized in this area of research tend to alter tissue architecture through dehydration and/or disruption of collagen fiber architecture [21, 24], limiting the function of these techniques for describing cell morphology in situ.

Within articular cartilage, chondrocytes show a gradient in morphology that starts with flattened disc-shaped (superficial zone) cells near the load-bearing surface, becoming more ovoid deeper within the bulk (middle zone) of the tissue [25]. Chondrocytes are enveloped by a pericellular matrix composed of type VI collagen and perlecan, and further surrounded with an interstitial ECM dominated by type II collagen fibrils and aggregates of hyaluronic acid and aggrecan. It has been traditionally thought that individual chondrocytes are isolated from direct contact with adjacent cells by these extensive ECM networks. Recently, researchers utilizing confocal microscopy to describe the 3D morphology of chondrocytes and the pericellular matrix proximal to the surface of the imaged tissue [25, 26] observed pronounced cytoplasmic extensions atypical of the homeostatic chondrocyte, which were correlated with osteoarthritis [27–29]. Additional reports on healthy cartilage have revealed apparent connectivity between adjacent cells based on cytoskeletal stains [30]. These previous confocal-based investigations of mapping the 3D distribution of chondrocytes have been limited to depths of less that 100 μm due to loss of signal in these dense tissues [28, 31], and attempts to characterize cellular morphology within the bulk of the tissue have necessitated mechanical sectioning, which can introduce artifacts. Our demonstration that SeeDB can enable visualization of chondrocytes at least 300 μm within intact tissues provides evidence of cell connectivity that may extend beyond the immediate pericellular space. Moreover, the novelty and utility of deep microscopy of chondrocytes is strengthened by our results from the membrane-specific DiI stain, confirming interactions between cells not only at the surface, but also at depths greater than 250 μm, far removed from any surface artifact (Fig. 3).

Of the cells associated with bone, osteocytes have proven to be the most difficult to image in situ due to their isolation within a dense type I collagen-hydroxyapatite matrix [32]. What is known about osteocyte morphology has come from studies that relied upon significant specimen processing such as decalcifying, dehydration, and/or cryosectioning to visualize cells below the surface [33] or estimated osteocyte shape using fluorescent probes to fill the lacunar space [34, 35]. Van Hoeve et al. visualized osteocytes within 60 μm-thick *z*-stacks; however, it was necessary for the images to be deconvolved to resolve any osteocyte processes [36]. After PFA fixation and clearing with SeeDB, osteocyte processes could be easily discerned 10–40 μm below the surface of intact cortical bone without extensive image processing (Fig. 6). Importantly, these high quality renderings of osteocyte processes were obtained using a mouse line in which tdTomato was localized to the plasma membrane, which were previously not apparent when imaging cryosections from the same mouse line [18, 37, 38]. By utilizing mouse lines engineered to have more intense fluorescence (e.g. ubiquitously expressed ZsGreen1 [17]) in combination with multiphoton microscopy, significant potential for enhanced depth penetration and resolution exists.

The 3D arrangement of cells within ligaments, tendons and menisci has received even less attention than that of cartilage and bone; however, that is not indicative of the importance of the role of these tissues within the musculoskeletal system. Ligaments and tendons are critical components that maintain the integrity of the articulated bony scaffold (via bone—ligament—bone linkages) and facilitate movement by transmitting muscle-generated force to bone. These tissues are primarily composed of aligned type I collagen fibrils embedded within a glycosaminoglycan- and proteoglycan-rich matrix, and have a very low cell density compared with most tissues. The unique arrangement of tendon cells within a 1D array, extending processes into the surrounding collagenous ECM was discovered through initial 3D investigations, but these studies were limited to serial sectioning with subsequent reconstruction [39] or imaging of 20 μm-thick cryosections [40]. Clearing of intact ligaments and tendons with SeeDB has the potential to reveal gradients in cell-cell interactions and morphology extending from the surface further into the bulk.

The menisci compensate for the geometric mismatch between opposing bones in certain joints, and facilitate the dissipation of energy and transfer of mechanical force across the joint space during loading and movement. Confocal microscopy of adult rabbit meniscus showed a graduation of cell morphology: stellate with long vimentin-rich processes at the surface to more rounded and fibrocartilagenous at the interface with articular cartilage [41]. While the stains employed within this study did not enable us to discern cells within the meniscus, the enhanced clearing afforded by SeeDB combined with the appropriate cytoskeletal antibodies will provide the opportunity to describe intercellular interactions across the meniscus.

This study purposefully utilized small molecular weight fluorescent probes and endogenously expressed fluorophores to efficiently test the utility of clearing ECM-rich tissues. While staining with antibodies enables the identification of specific molecules of interest, the larger molecular weight introduces long incubation times (10 days—6 weeks) to stain structures deep within the tissue of interest and requires high concentrations of antibodies [3, 42]. We are currently working on optimizing staining conditions to visualize the distribution of specific ECM.

Our data demonstrate how optical clearing can enhance the study of cellular morphology in ECM-rich tissues. Adaptation of optical clearing methods such as SeeDB will further the knowledge of intercellular interactions and tissue structure within the intact musculoskeletal system, critical to the understanding of recapitulation strategies of regenerative medicine and tissue engineering. We anticipate that optical clearing of connective tissues will find application in a broad range of applications in physiology and pathology.

## Supporting Information

S1 Fig Optical clearing of musculoskeletal tissues. Equilibration of bovine knee tissues to SeeDB substantially enhanced the macroscopic transmission of light though muscle (m) and tendon (t, top row) and synovium (bottom row). Control and cleared samples were imaged using the same acquisition parameters on a Leica MZ80 stereomicroscope. Grid spacing = 2.1 mm.
(TIF)

S2 Fig Confocal visualization of cellular and tissue architecture is enhanced at greater depths after optical clearing. **A—D’:** Bovine ligament stained with WGA (green) and Hoescht 34580 (blue). **E—H’:** Bovine cartilage stained with WGA only. In controls, fluorescence intensity of WGA greatly diminished 25 μm into the samples. After clearing, the ECM architecture could be easily visualized in both ligament and cartilage at 25 μm. **I—L’**: Chondrocytes stained with phalloidin (green) and Hoescht 34580 could barely be visualized 50 μm deep, whereas after clearing the actin filaments and nuclei could be seen throughout the entire 212 μm × 212 μm × 100 μm representative volume element. Specimens were imaged using a Nikon A1R microscope, with a PlanFluor 20× multi-immersion objective, NA = 0.75, 3× optical zoom and 2× frame average. For control samples, water was the immersion medium and cleared samples utilized oil with a refractive index of 1.515. Image dimensions: 512 × 512 pixels^2^, Δ*z* = 1.0 μm. Stacks were rendered in 3D using FIJI. Bars in A, D, E, H, I and L = 50 μm.
(TIF)

S1 Movie 3D rendering of bovine cartilage stained with DiI from Fig. 3. The image stack was acquired using a Nikon A1R microscope, with a PlanFluor 20× multi-immersion objective (oil *n* = 1.515), NA = 0.75, 3× confocal zoom and 2× frame average. Image dimensions = 212 × 212 μm2, 512 × 512 pixels^2^, Δ*z* = 1.0 μm, 307 slices. The image stack rendered in 3D using FIJI.
(AVI)

S2 Movie 3D rendering of intercondrocyte communication near the articular surface taken from the image stack shown in M1 and Fig. 3. Image acquisition parameters were provided in M1, except: image dimensions were 35 × 44 × 29 μm^3^, Δ*z* = 1.0 μm.
(AVI)

S3 Movie 3D rendering of a chondrocyte pair shown in Fig. 3. Image acquisition parameters were provided in M1, except: image dimensions were: 26 × 30 × 13 μm^3^, Δ*z* = 1.0 μm.
(AVI)

S4 Movie 3D rendering of an intercondrocyte communication 265 μm away from articular surface taken from stack shown in M1 and Fig. 3. Image acquisition parameters were provided in M1, except: image dimensions were 24 × 37 × 21 μm^3^, Δ*z* = 1.0 μm.
(AVI)
