# Supplementary figures and images for: Optical Clearing in Dense Connective Tissues to Visualize Cellular Connectivity In Situ
Source: PLoS One. 2015 Jan 12;10(1):e0116662. doi: 10.1371/journal.pone.0116662 (PMC4291226; doi:10.1371/journal.pone.0116662)

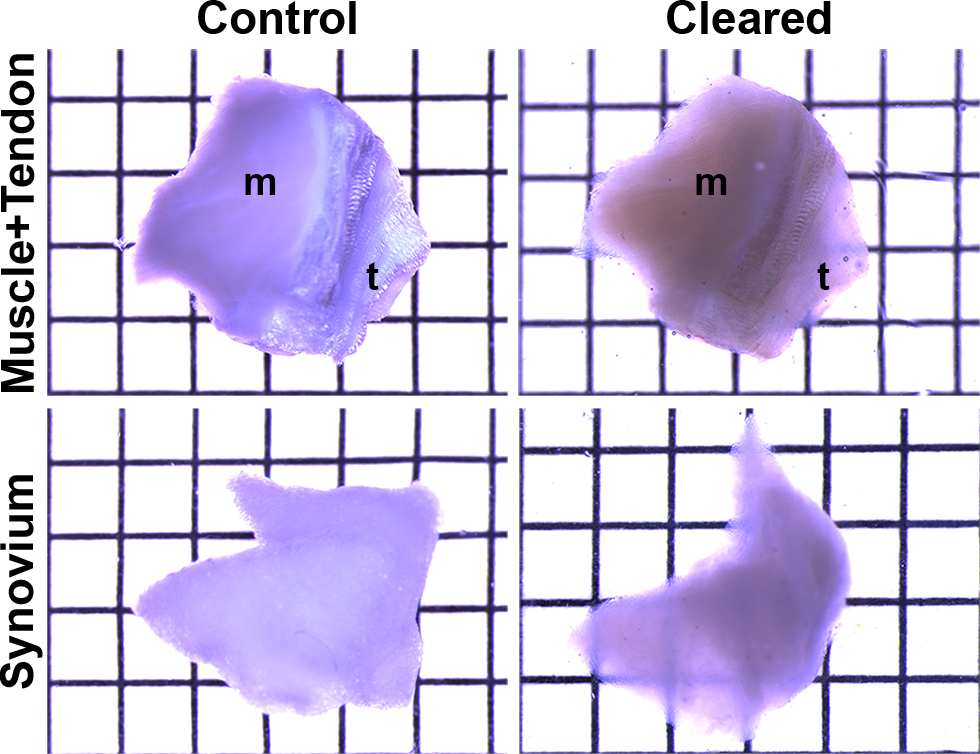

Supplement: S1 Fig — Equilibration of bovine knee tissues to SeeDB substantially enhanced the macroscopic transmission of light though muscle (m) and tendon (t, top row) and synovium (bottom row). Control and cleared samples were imaged using the same acquisition parameters on a Leica MZ80 stereomicroscope. Grid spacing = 2.1 mm. (TIF) [file pone.0116662.s001.tif]

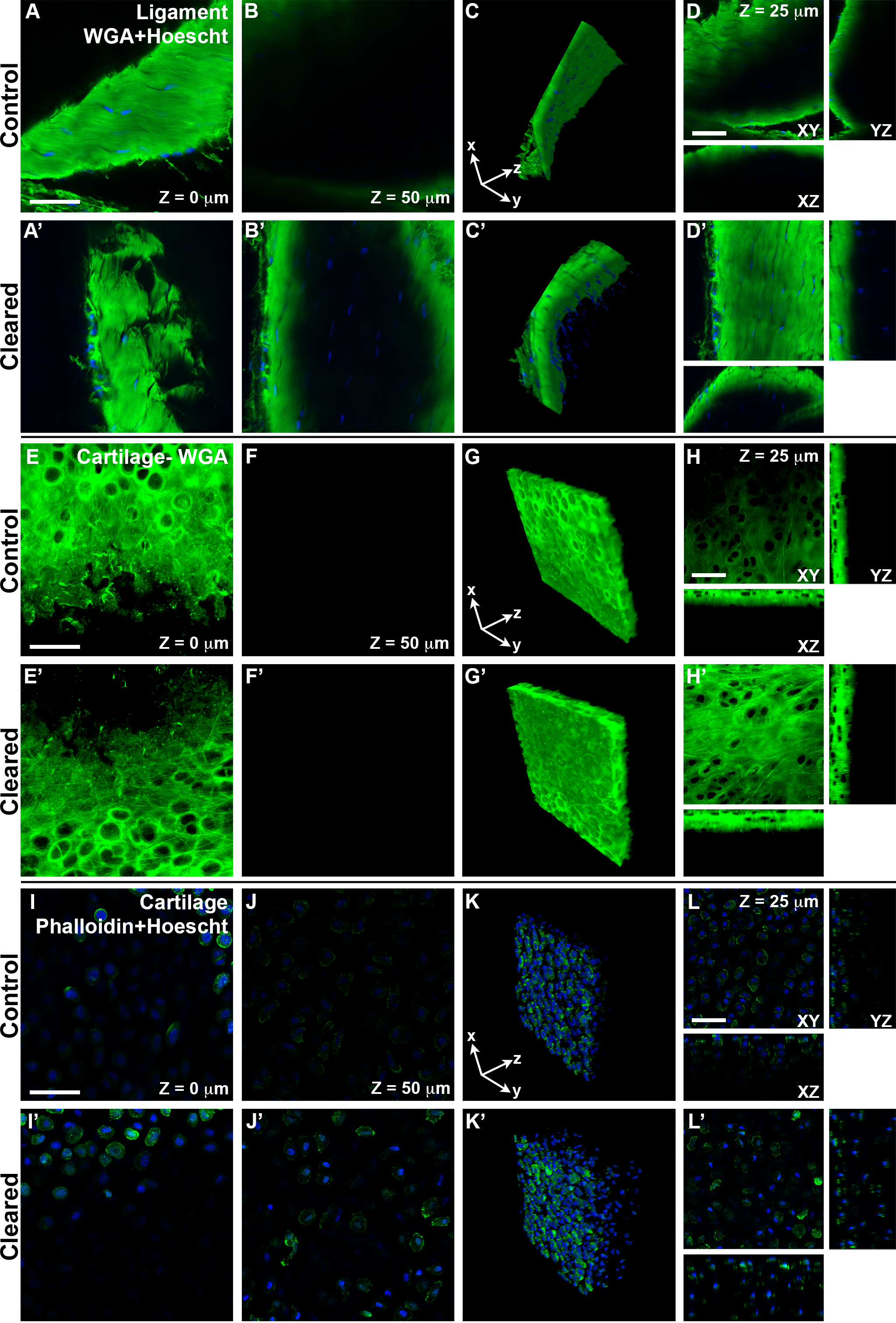

Supplement: S2 Fig — A—D’: Bovine ligament stained with WGA (green) and Hoescht 34580 (blue). E—H’: Bovine cartilage stained with WGA only. In controls, fluorescence intensity of WGA greatly diminished 25 μm into the samples. After clearing, the ECM architecture could be easily visualized in both ligament and cartilage at 25 μm. I—L’: Chondrocytes stained with phalloidin (green) and Hoescht 34580 could barely be visualized 50 μm deep, whereas after clearing the actin filaments and nuclei could be seen throughout the entire 212 μm × 212 μm × 100 μm representative volume element. Specimens were imaged using a Nikon A1R microscope, with a PlanFluor 20× multi-immersion objective, NA = 0.75, 3× optical zoom and 2× frame average. For control samples, water was the immersion medium and cleared samples utilized oil with a refractive index of 1.515. Image dimensions: 512 × 512 pixels2, Δz = 1.0 μm. Stacks were rendered in 3D using FIJI. Bars in A, D, E, H, I and L = 50 μm. (TIF) [file pone.0116662.s002.tif]
